# Dietary oleic acid regulates hepatic lipogenesis through a liver X receptor-dependent signaling

**DOI:** 10.1371/journal.pone.0181393

**Published:** 2017-07-21

**Authors:** Simon Ducheix, Alexandra Montagner, Arnaud Polizzi, Frédéric Lasserre, Marion Régnier, Alice Marmugi, Fadila Benhamed, Justine Bertrand-Michel, Laila Mselli-Lakhal, Nicolas Loiseau, Pascal G. Martin, Jean-Marc Lobaccaro, Laurent Ferrier, Catherine Postic, Hervé Guillou

**Affiliations:** 1 INRA, ToxAlim, Toulouse, France; 2 Université de Toulouse, INP, UPS, ToxAlim, Toulouse, France; 3 INSERM, U1016, Institut Cochin, Paris, France; 4 CNRS, UMR8104, Paris, France; 5 MetaToul, Plateau de lipidomique, INSERM, Toulouse, France; 6 Clermont Université, Université Blaise Pascal, Génétique Reproduction et Développement, Clermont-Ferrand, France; 7 CNRS, UMR 6293, GReD, Aubière, France; 8 INSERM, U1103, GReD, Aubière, France; 9 Centre de Recherche en Nutrition Humaine d’Auvergne, Clermont-Ferrand, France; 10 Université Paris Descartes, Sorbonne Paris Cité, Paris, France; Nihon University School of Medicine, JAPAN

## Abstract

Olive oil consumption is beneficial for health as it is associated with a decreased prevalence of cancer and cardiovascular diseases. Oleic acid is, by far, the most abundant component of olive oil. Since it can be made through *de novo* synthesis in animals, it is not an essential fatty acid. While it has become clear that dietary oleic acid regulates many biological processes, the signaling pathway involved in these regulations remains poorly defined. In this work we tested the impact of an oleic acid-rich diet on hepatic gene expression. We were particularly interested in addressing the contribution of Liver X Receptors (LXR) in the control of genes involved in hepatic lipogenesis, an essential process in whole body energy homeostasis. We used wild-type mice and transgenic mice deficient for both α and β Liver X Receptor isoforms (LXR-/-) fed a control or an oleate enriched diet. We observed that hepatic-lipid accumulation was enhanced as well as the expression of lipogenic genes in the liver of wild-type mice fed the oleate enriched diet. In contrast, none of these changes occurred in the liver of LXR-/- mice. Strikingly, oleate-rich diet reduced cholesterolemia in wild-type mice and induced signs of liver inflammation and damage in LXR-/- mice but not in wild-type mice. This work suggests that dietary oleic acid reduces cholesterolemia while promoting LXR-dependent hepatic lipogenesis without detrimental effects to the liver.

## Introduction

Epidemiological studies show that consumption of olive oil rich diet, and more generally Mediterranean diet, in which the main component is olive oil, is associated with longevity and a decreased prevalence of coronary heart diseases, cancers and metabolic syndrome (reviewed in [1]). The major fatty acid provided by olive oil is oleic acid or oleate (C18:1 n-9) and it is known to contribute to the beneficial effects of olive oil consumption [2]. Recently, it has been shown that dietary oleic acid influences C. Elegans lifespan [3]. However, little is known about the signaling pathways that may be sensitive to high dietary oleate.

In this work, we aimed at investigating the effects of dietary oleic acid on the regulation of hepatic gene expression. Indeed, while oleic acid can be synthesized *de novo* through the activity of the Stearoyl-CoA desaturase 1 (SCD1), it is clear from results obtained in germline *Scd1* KO mice [4–7], liver-specific *Scd1* KO mice [8;9] and in mice overexpressing *Scd3* [10] that oleate can contribute to various physiological functions, notably in liver. Dietary olive oil [11] and oleic acid [12] have been shown to have beneficial effects in different experimental models of hepatic pathologies termed Non Alcoholic Fatty Liver Diseases (NAFLD) that range from steatosis to steatohepatitis (NASH). Despite its promoting effect on hepatic lipid accumulation, oleic acid synthesized *de novo* also contributes to protect hepatocytes from insulin resistance [13].

Recently, it has been shown that oleic acid could modulate the activity of Liver X Receptors (LXR) in human neutrophils [14]. Moreover, LXR activity is sensitive to fatty acids *in vitro* [15] and it plays a central role in the hepatic effect of dietary fatty acids on lipogenesis *in vivo* [16]. The LXRs are class II nuclear receptors [17]. They are sensitive to oxidized cholesterol derivatives, the oxysterols, which bind to and activate both LXR isotypes (α, NR1H3 and β, NR1H2). A rise in oxysterol concentration triggers transcription of LXR target genes. For instance, LXRα and β regulate genes involved in lipogenesis [18]. LXRα binds to promoters of lipogenic genes such as *Fasn* (Fatty acid synthase) [19] or *Scd1* [20] and directly promote *de novo* fatty acid synthesis. Therefore, pharmacological activation of LXR leads to hepatic neutral lipid accumulation that is the hallmark of NAFLD [21]. LXRs are also involved in the reverse cholesterol transport and cholesterol degradation into bile acids. LXR is not only involved in the excretion of cholesterol [22] but also in the repression in inflammation [23] and, thereby, may be involved in protecting the liver from insults that may occur in NAFLD [24].

In this study, we investigated the contribution of LXR to the modulation of lipogenesis, cholesterol metabolism and inflammation by dietary oleic acid in the liver. We used a nutritional approach in wild-type mice and in transgenic mice lacking the two LXR isoforms (LXR-/-). We demonstrated that LXR is required for the lipogenic genetic response as well as the decreased cholesterolemia in response to a diet providing a high content of oleic acid. Moreover, we identified that, in this process, LXR is protective against lipogenesis-induced liver inflammation and damage. This work reveals for the first time that LXR contributes to the effects induced by dietary oleic acid and protects liver from inflammation while inducing lipogenesis.

## Materials and methods

### Animals and diets

Eight week-old males LXRα β double deficient mice and their wild-type counterpart with a mixed C57BL6/129SvJ genetic background were fed *ad libitum* for 9 weeks a reference diet (REF) or an oleate-rich diet (OLIV) (pellets prepared by UPAE-INRA, Jouy-en-Josas, France, replaced twice a week) with free access to water. Diets were isocaloric and contain 5% fat (w/w). Oils used for experimental diet preparation were grape seed and colza oils (50/50) for the REF diet and olive oil for the OLIV diet. The composition of the diets and oils are given in S1 and S2 Tables, respectively. Mice were sacrificed at ZT2. All mice were bred at INRA’s transgenic rodent facility at 22 ± 2°C. The animal facilities used are licensed by the relevant local authorities for rodents (agreement C31 555 13). All animal experiments were performed in accordance with the guidelines of European legislation (Council Directive 2010/63/UE) and French Decree 2013–118 on the protection of animals used for scientific purposes and were approved by the Local Animal Care and Use Committee (TOXCOM-133) and French Ministry of Higher Education and Research (agreement CEEA-86).

### Blood and organ sampling

Blood was collected at the submandibular vein in heparin-coated capillaries. Plasma was prepared by centrifugation (1500×g, 10 min) and kept at -80°C until use. Following euthanasia, tissues were removed, weighed, dissected, snap-frozen in liquid nitrogen and stored at -80°C until use.

### Gene expression studies

Total RNA was extracted with TRIzol^®^ reagent (Invitrogen). For real-time quantitative PCR (qPCR), total RNA samples (2 μg) were reverse-transcribed using High Capacity cDNA Reverse Transcription Kit (Applied Biosystems). Primers for SYBR Green assays are presented in S3 Table. Amplifications were performed on an ABI Prism 7300 Real Time PCR System (Applied Biosystems). QPCR data were normalized by TATA-box binding protein (*Tbp*) mRNA levels and analyzed with LinRegPCR.

### Immunoblot analysis

Protein extracts were prepared using the Proteo-Jet cytoplasmic and nuclear extraction kit (Fermentas). Following separation by SDS-PAGE liver proteins were probed with primary antibodies from Cell Signaling (β-ACTIN: 4970; LAMIN A/C: 2032; ACLY: 4332; ACLY-P: 4331; ACC: 3662; FASN: 3189), Abcam (ELOVL6: 69857), Santa Cruz Biotechnology (SCD1: sc-14719; LXR: sc-13068), Lab Vision (SREBP-1: MS-1207-P1ABX), Novus Biological (ChREBP: nb400-135) and secondary antibodies from Biotium (CF680 or CF770-labeled). The images were analyzed on the Odyssey Infrared Imaging system (LI-COR Biosciences).

### Biochemical assays

Hepatic lipid content, fatty acids composition and plasma biochemistry was performed as described earlier [25].

### Histology

Frozen liver samples were embedded in Neg 50 (Fisher Scientific). Sections (5 μm, Leica RM2145 microtome) were stained with Oil- Red-O and visualized with a Leica DFC300 camera (Leica).

### Statistical analysis

All data were analyzed using R (www.r-project.org). Data are expressed as the mean ± SEM. Differential effects were analyzed by Anova followed by Student t-tests with a pooled variance estimate. For genes expression analysis, p-values were adjusted according Benjamini-Hochberg procedure. A p-value ≤ 0.05 was considered significant.

## Results

### LXR deficiency prevents hepatic lipid accumulation in response to dietary oleate

**Fig 1:**
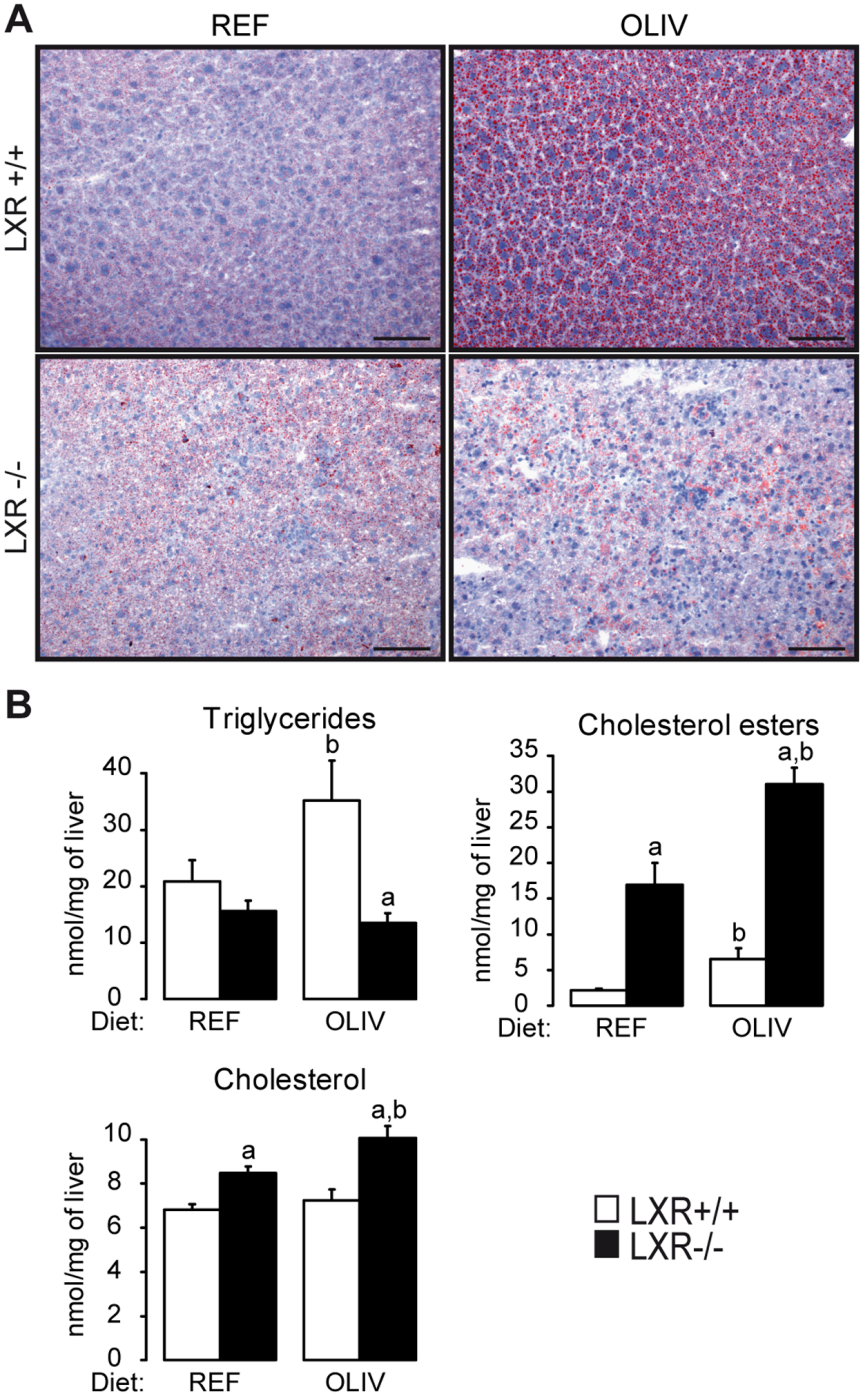
High oleic acid diet induces hepatic steatosis in LXR+/+ but not in LXR-/- mice. (A) Representative Oil Red O-stained frozen sections of liver from mice of both genotypes fed the REF or the OLIV diet (Scale bars: 100 μm). Neutral lipids appear in red. (B) Liver triglycerides, cholesterol and cholesterol esters measured by gas chromatography. Data are the mean±SEM of values measured in LXR+/+ and LXR-/- mice fed the REF or the OLIV diet. ^a^ Significant genotype effect. ^b^ Significant difference versus REF diet (n = 6 mice per group).

In order to investigate whether LXR may be involved in the lipogenic response to oleate, we tested the effects of dietary oleic acid *in vivo*. A reference diet (REF, 40% oleic acid) and an oleate rich diet (OLIV; 79% oleic acid) were given for 9 weeks to wild-type and LXR-/- mice. The relative composition of hepatic fatty acids was measured (Table 1). As expected we observed an enrichment of oleic acid in the liver of wild-type and LXR-/- mice fed the OLIV diet compared to mice fed the REF diet. The diet and the genotype did not impact body weight, insulinemia or glycemia (Table 2). Wild-type mice fed the OLIV diet displayed lower perigonadic white adipose tissue weight compared to mice fed the REF diet. Fatty acid composition of this tissue was also altered. Indeed, in the wild-type mice OLIV diet tended to decrease n-6/ n-3 ratio compared with the REF one (S4 Table). No modification occurred in LXR deficient mice for both white adipose weight and its n-6/ n-3 ratio (Table 2 and S4 Table). Interestingly, liver weight was increased by the OLIV diet in both genotypes (Table 2). However, the OLIV diet led to neutral lipids accumulation in the liver of wild-type mice, which was not observed in mice lacking LXR (Fig 1A). Quantification of hepatic cholesterol, cholesterol esters and triglycerides showed that while cholesterol esters are increased in both genotypes, OLIV diet leads to higher levels of triglycerides only in wild-type mice (Fig 1B). Interestingly, LXR-/- mice fed the OLIV diet showed an increase in free cholesterol while this did not occur in wild-type mice (Fig 1B). Altogether, these results suggest that the oleic acid-rich diet induces liver steatosis in a LXR-dependent manner.

**Table 1:** Effects of an oleic acid-rich diet on hepatic fatty acid profile in LXR+/+ and LXR-/- mice.

	LXR+/+		LXR-/-	
	REF	OLIV	REF	OLIV
C14:0	0,263	0.488^b^	0.237	0.169^a^
C16:0	22,53	22.93	18.516^a^	16.280^a^
C16:1 n-9	0.791	1.056	0.767	1.306
C16:1 n-7	3.385	4.805	4.977^a^	5.342
C18:0	12.057	10.704	13.81	11.947
C18:1 n-9	16.605	32.629^b^	14.215	28.523^b^
C18:1 n-7	3.578	6.128^b^	4.425	6.787b
C18:2 n-6	15.968	5.450^b^	20.862^a^	10.809^a^^,^^b^
C18:3 n-6	0.383	0.355	0.352	0.416
C18:3 n-3	0.313	0.121^b^	0.663^a^	0.126^b^
C20:1 n-9	0.383	0.563^b^	0.477	0.792^a^^,^^b^
C20:2 n-6	0.393	0.000^b^	0.426	0.000^b^
C20:3 n-9	0.000	1.584^b^	0.000	1.627^b^
C20:3 n-6	1.516	1.616	1.569	1.45
C20:4 n-6	12.859	6.360^b^	10.906	8.438
C20:3 n-3	0.133	0.067	0.315^a^	0.185
C20:5 n-3	0.281	0.053	0.396	0.326
C22:4 n-6	0.214	0.196	0.282	0.380^a^
C22:5 n-6	0.51	0.812^b^	0.234^a^	0.507^a^^,^^b^
C22:5 n-3	0.263	0.794^b^	0.502	0.233^a^
C22:6 n-3	7.202	2.753^b^	5.78	2.966^b^
C24:1 n-9	0.373	0.537	0.291	1.393^b^
n-6/ n-3	4,059	4,326	4,601	5,742^a^^,^^b^
PUFAs/MUFAs	1,700	0,509^b^	1,837	0,657^b^

Fatty acids were analyzed by gas chromatography (n = 5 animals per group). Data are the mean of the masse percentage measured in liver of LXR+/+ and LXR-/- mice fed the REF or the OLIV diet.

^a^ Significant genotype effect.

^b^ Significant difference versus REF diet.

**Table 2:** Effects of an oleic acid-rich diet on body, liver, adipose tissue weights, and on plasma parameters in LXR+/+ and LXR-/- mice.

	LXR+/+		LXR-/-	
	REF	OLIV	REF	OLIV
Body weight (g)	31.4	29.9	29.8	29.4
Liver weight % (w/w)	3.77	4.51^b^	3.58	4.6^b^
pWAT weight % (w/w)	3.71	2.55^b^	3.43	3.04
Plasma insulin (ng/mL)	1.379	1.41	1.112	0.881
Plasma glucose (mmol/L)	8.36	8.082	8.607	8.763
Plasma triglycerides (mmol/L)	1.21	1.04	0.883	0.598^a^^,^^b^
Plasma VLDL-TG (mmol/L)	0.859	0.725	0.883	0.598

Body weight gain, liver weight (somatic index), perigonadic white adipose tissue (pWAT) weight (somatic index) and plasma insulin, glucose, triglycerides, VLDL-TG of LXR+/+ and LXR-/- mice fed the REF or the OLIV diet. Data are the mean±SEM.

^a^ Significant genotype effect.

^b^ Significant difference versus REF diet (n = 6 mice per group).

### LXR is important in the modulation of hepatic gene expression in response to an oleate rich diet

**Fig 2:**
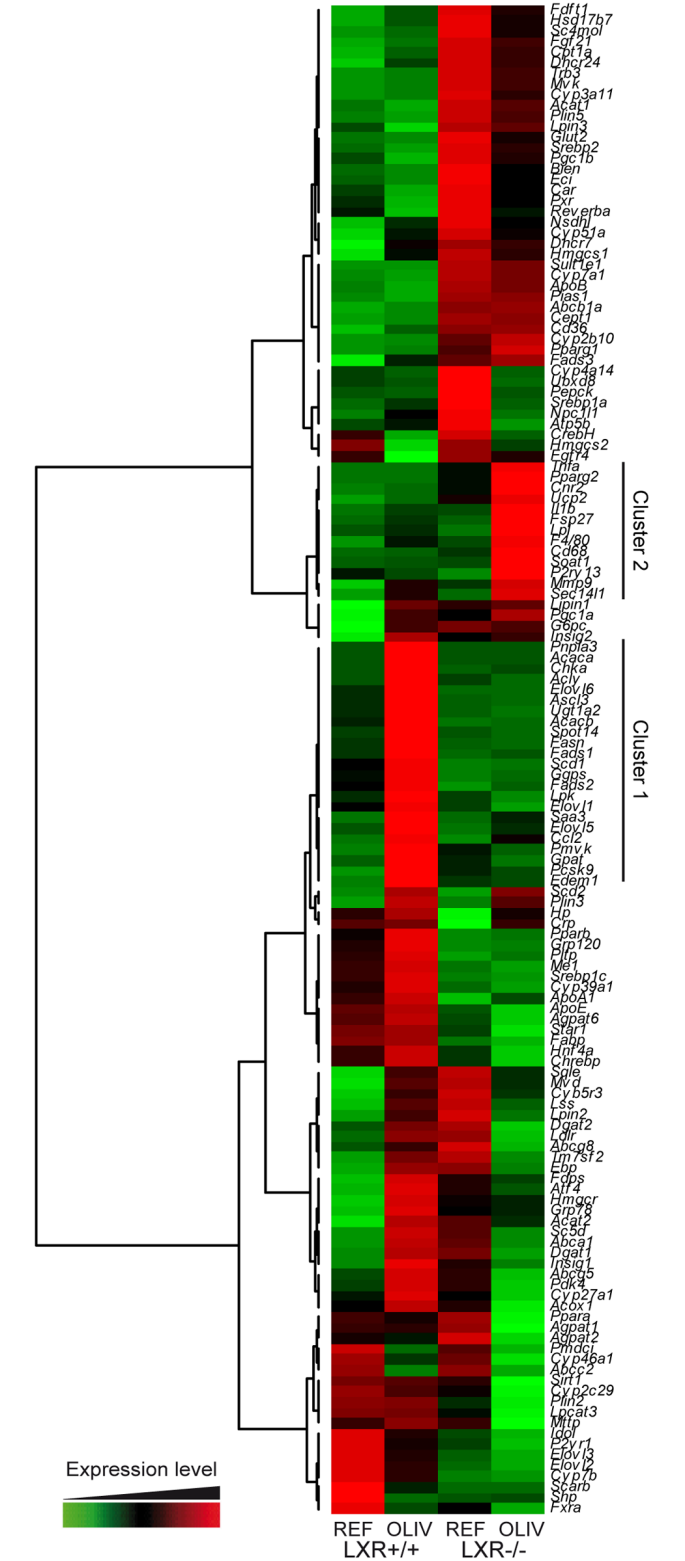
High oleic acid diet modulates hepatic gene expression. Hepatic gene expression of 142 genes related to lipid metabolism, nuclear receptor signaling and inflammation were quantified by qPCR from liver of LXR+/+ and LXR-/- mice fed the REF or the OLIV diet. Data are presented as a heatmap associated with a hierarchical classification.

To further investigate the role of LXR, we next measured the hepatic expression of a set of 142 genes involved in lipid metabolism, nuclear receptors signaling and inflammation (Fig 2). Results were illustrated in a heatmap coupled with hierarchical classification, which revealed clusters of genes differently modulated by OLIV diet depending on the genotype. The first cluster (Cluster 1) is composed of genes specifically up-regulated in wild-type mice fed the OLIV diet compared to the REF group as this regulation did not occur in LXR-/- mice (Fig 2). This cluster contained genes involved in lipogenesis such as *Acly*, *Acaca*, *Fasn*, *Elovl6* and *Scd1*. The heatmap also highlighted a cluster of genes (Cluster 2) whose expression is not modified in wild-type mice but increased in LXR-/- mice fed the OLIV diet (Fig 2). This cluster mostly relates to genes involved in inflammatory processes such as *Tnf*α, *Il1β* and *F4/80*. These two clusters we identified shed the light on the role of LXR in uncoupling lipogenesis and inflammation. As these two molecular processes are known to be both important in NAFLD development we further investigated these pathways in our *in vivo* model.

### LXR deficiency impairs the lipogenic response to an oleate-rich diet

**Fig 3:**
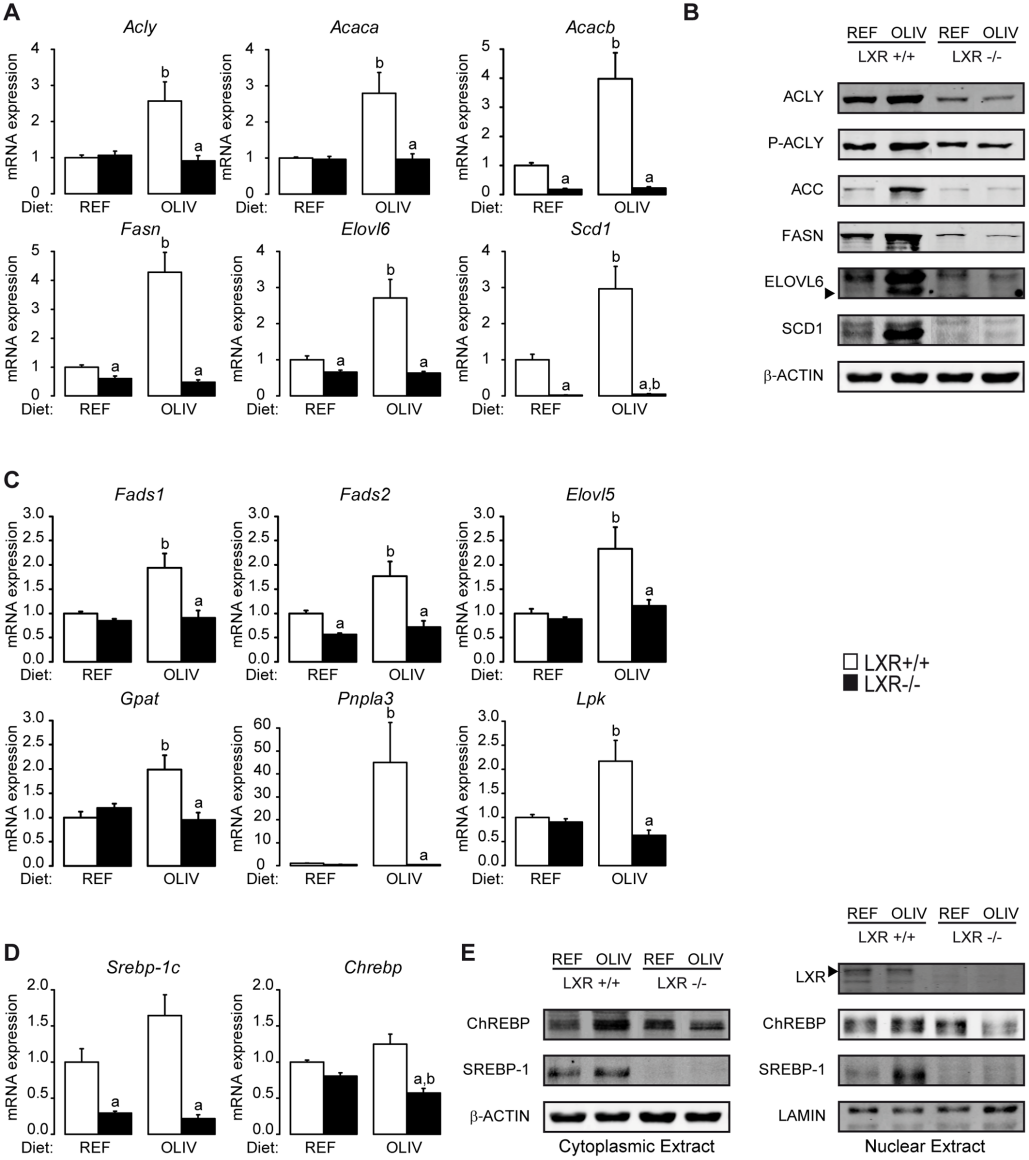
LXR mediate the induction of lipogenesis induced by an oleic acid-rich diet. (A) Hepatic *Acly*, *Acaca*, *Acacb*, *Fasn*, *Elovl6*, *Scd1* mRNA levels quantified by qPCR. (B) Cytoplasmic protein expression levels of P-ACLY, ACLY, ACC, ELOVL6, SCD1, FASN AND β-ACTIN assayed by Western Blotting. (C) *Fads1*, *Fads2*, *Elovl5*, *Gpat*, *Pnpla3* and *Lpk* mRNA quantification assayed by qPCR. (D) *Srebp-1c* and *Chrebp* mRNA quantification assayed by qPCR. (E) Cytoplasmic and nuclear expression levels of LXR, SREBP-1c and ChREBP assayed by Western Blotting. Data are the mean±SEM of values measured in LXR+/+ and LXR-/- mice fed REF or OLIV diet. ^a^ Significant genotype effect. ^b^ Significant difference versus REF diet (n = 6 mice per group).

The OLIV diet increased the expression of genes involved in lipogenesis such as *Acly*, *Acaca*, *Acacb*, *Fasn*, *Elovl6* and *Scd1* in an LXR dependent manner (Fig 3A). In order to further investigate this regulation, the expression of key lipogenic proteins was measured (ACLY, its active phosphorylated form P-ACLY, ACC, FASN, ELOVL6 and SCD1) (Fig 3B: S1 Fig). Protein levels were consistent with the LXR-dependent changes observed in mRNA. In addition to genes related to lipogenesis, the cluster 1 also contains genes involved in fatty acids desaturation (*Fads1* and *Fads2*) and elongation (*Elovl5*). Expression of those genes was also induced by OLIV diet in an LXR-dependent manner. Importantly, this correlates with the concomitant enrichment of C22:5n-3 which only occurred in the liver of wild-type mice fed the OLIV diet (Table 1). This cluster also contains genes involved in glycolysis (*Lpk*) and in triglycerides synthesis and remodeling (*Gpat* and *Pnpla3*) which displayed the same expression pattern as lipogenic genes (Fig 3C). To further investigate the role of oleic acid on hepatic lipogenesis, we studied the implication of the Sterol Responsive Element Binding Protein-1c (SREBP-1c) and the Carbohydrate Response Element Binding Protein (ChREBP), two transcription factors which are major regulators of lipogenesis in liver, and whose activation is reflected by their nuclear localization. Although no change was observed at the gene expression level in response to the OLIV diet in LXR+/+ mice, oleic acid-rich diet induced an LXR-dependent increase in SREBP-1c nuclear expression, suggesting that oleate modulates lipogenesis via a pathway involving an LXR-dependent SREBP-1c activation (Fig 3D and 3E; S1 Fig).

### LXR deficiency reveals hepatic inflammation in mice fed an oleate-rich diet

**Fig 4:**
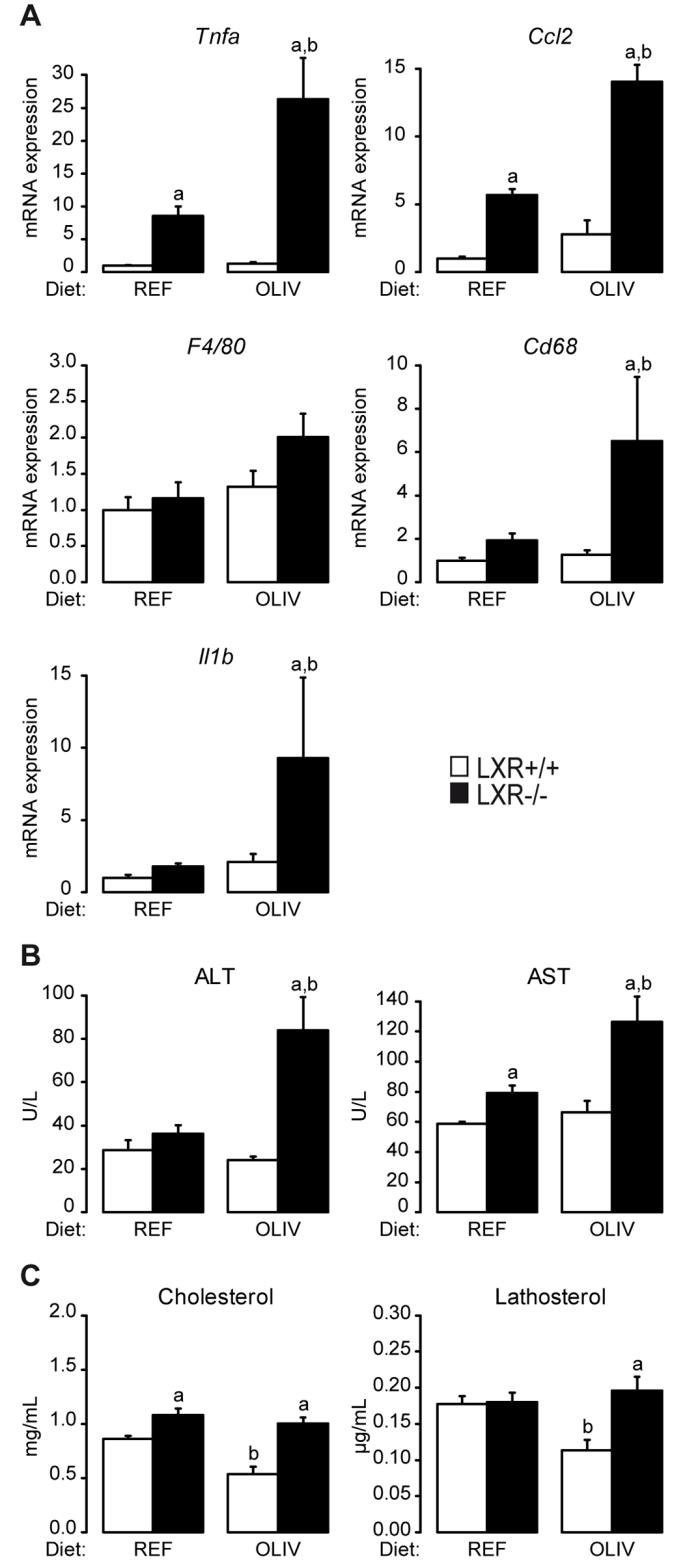
LXR protects from hepatic damage induced by an oleic-rich diet. (A) *Tnf*α, *Ccl2*, *F4/80*, *Cd68* and *Il1β* mRNA quantification assayed by qPCR. (B) Plasma activity of ALT and AST. (C) Plasma cholesterol and lathosterol levels analyzed by gas chromatography. Data are the mean±SEM of values measured in LXR+/+ and LXR-/- mice fed the REF or the OLIV diet. ^a^ Significant genotype effect. ^b^ Significant difference versus REF diet (n = 6 mice per group).

The second cluster contains several genes involved in inflammation (Fig 2). In agreement with the anti-inflammatory properties of LXR, we observed an increase of *Tnf*α and *Ccl2* in LXR-/- mice compared to wild-type controls fed the REF diet. However, all the inflammatory genes of this cluster (*Tnf*α, *Ccl2*, *F4/80*, *Cd68* and *Il1β*) displayed no diet modulation in wild-type mice, although, in LXR-/- animals, the OLIV diet increased their expression (Fig 4A). To measure possible hepatic damages we assayed plasmatic activity of alanine aminotransferase (ALT) and aspartate aminotransferase (AST). In wild-type mice the OLIV diet did not trigger any modification in the concentration of these two enzymes compared to mice fed the REF diet. However, in LXR-/- animals, the OLIV diet increased ALT and AST concentration compared to the REF diet (Fig 4B). Hepatic n-3 and n-6 fatty acids composition as well as the n-6/ n-3 ratio have been shown to be associated with NASH [26;27]. In our model, OLIV diet feeding increased eicosapentaenoic acid (C20:5 n-3) proportion in wild-type mice although in LXR-/- animals this diet was decreasing it. Inversely, OLIV diet reduced C18:2 n-6 concentration in a lesser extent in LXR-/- animals compared to the wild-type ones. Similarly, C20:4 n-6 content was reduced in the liver of wild-type mice fed the OLIV diet compared to the REF diet fed ones. In the LXR-/- mice this regulation was lost. More generally, the hepatic n-6/ n-3 ratio was not undergoing a dietary regulation in the wild-type mice but in the LXR-/- animals this ratio was increased by the OLIV diet compared to the REF one (Table 1).

Free cholesterol has been identified as a potent inflammatory signal [28;29]. We have noticed that the hepatic content of free cholesterol (Fig 1B) displays the similar regulation than the inflammatory genes assayed in the liver as well as ALT and AST plasmatic activity (Fig 4B). Moreover, we observed that the OLIV diet decreases both plasma cholesterol and lathosterol, an indicator of whole body cholesterol synthesis, compared to the REF diet in wild-type mice. In mice lacking LXR no modification occurred (Fig 4C). Therefore, when mice are fed an oleate-rich diet, LXR contributes to decrease plasma cholesterol concentrations while its deficiency unravels signs of hepatotoxicity in response to OLIV diet.

## Discussion

The Liver X Receptors are type II nuclear receptors which are involved in cholesterol reverse transport, its excretion through the bile [22] or trans-intestinal cholesterol efflux [30;31] and in the limitation of inflammation [32] and proliferation [33]. As a consequence, LXR has emerged as potent target to treat large range of diseases related to cholesterol from cardiovascular diseases such as atherosclerosis [34] to cancer [35] or Alzheimer’s disease [36]. However, these promising properties are hampered by the fact that LXR is a potent activator of key genes involved in the control of hepatic lipogenesis. Indeed, LXRE have been described in *Acaca* [37], *Fasn* [19], *Scd1* [20], *Srebp-1c* [38] and *Chrebp* [39] promoters. Moreover, LXR is important for the activity of SREBP-1c [40] and ChREBP [41] and its activation by synthetic agonist induces lipogenesis, steatosis and VLDL secretion [21].

Dietary oleic acid exerts several beneficial effects in many organs linked to energy homeostasis. Dietary supplementation with an oleic acid-derived compound restores gut microbiota dysbiosis induced by high fat diet [42]. Oleate supplementation in high fat diet increases anti-inflammatory M2 macrophages [43] and improves insulin signaling and inflammatory parameters [44] in white adipose tissue. In skeletal muscle cells, oleate signaling triggers fatty acid oxidation through a PKA-SIRT1-PGC1α axis [45]. It has been recently published that oleic acid, the major fatty acid present in olive oil, could modulate the expression of LXR target genes in human neutrophils [46]. Observational studies show that a correlation exists between olive oil consumption and a decreased prevalence of cardiovascular diseases and cancers [1] which are linked to LXR activity.

In this study, we questioned whether LXR could mediate the effects of dietary oleic acid. As it has been described that this fatty acid increases lipogenesis and triglycerides accumulation in the liver [9], we focused on hepatic lipid metabolism and in particular on lipogenesis. To address this issue, we used wild-type and knockout mice lacking both LXR isoforms that were fed either a control diet (40% oleate) or an oleic acid-rich diet (79% oleate). When challenged with these two isocaloric diets, both wild-type and LXR-/- mice displayed major changes in hepatic fatty acids composition (Table 1). However, while hepatic triglyceride content of wild-type mice was increased, LXR-/- mice livers displayed no changes in response to the diet rich in oleic acid (Fig 1). Moreover, by measuring the hepatic expression of 142 genes involved in energy metabolism, nuclear receptor signaling and inflammation, we identified a cluster of genes following the same modulation pattern as hepatic triglycerides (Fig 2). In wild-type mice, high oleic acid diet stimulated lipogenic gene expression and protein content in liver in a LXR-dependent manner. The oleic acid-rich diet had no effects on nuclear ChREBP accumulation but promoted SREBP-1c activation in a LXR-dependent way (Fig 3). In vitro we did not observe any regulation of lipogenic genes in response to oleic acid on the cell lines we tested (S2 Fig). However, when we compared the effect of triolein to olive oil in vivo we observed an increase in lipogenic gene expression in response to triolein. Altogether, these data suggest that LXR is required for the hepatic response to oleic acid in vivo.

Although it was reported that LXR activation by a synthetic ligand triggers lipogenesis and VLDL-TG secretion [21], in our study the LXR mediated up-regulation of lipogenesis by the OLIV diet was not associated with hypertriglyceridemia (Table 2), or high levels of VLDL-TG (Table 2) which is observed in mice treated with potent pharmacological agonist of LXR. It has been demonstrated that long-term treatment with an LXR agonist has beneficial effects on adipose tissue homeostasis, notably by mitigating inflammatory parameters, decreasing visceral adipose tissue weight and redistributing fat from visceral adipose tissue to subcutaneous adipose tissue [46]. Although we did not focus on adipose tissue, we observed an LXR-dependent decrease of perigonadic adipose tissue weight (Table 2) associated with a modulation of fatty acid composition reflecting a lower inflammatory status in mice fed the OLIV diet (S4 Table). These data are in agreement with the work of Archer *et al*. in which long term LXR activation leaded to fat redistribution from visceral to subcutaneous fat pads and reduction of inflammation in these tissues [46].

The most common model of progression from NAFLD to NASH is a two-step model. The first hit is the accumulation of triglycerides in the liver or steatosis. The second step encompasses several hepatic injuries including inflammation and cholesterol overloading. However, this classic model has been challenged (reviewed in [47]). Here, we provide new model of dissociation between steatosis and hepatic damage. Indeed, through our gene expression analysis in the liver, we identified a cluster of genes involved in inflammation whose expression does not change in wild-type mice but increases in LXR knockout mice fed the oleic acid-rich diet (Fig 4A). This cluster contains genes encoding for TNF-α and IL-1β whose expression is associated with the one of CXCL10, a newly described marker of NASH [48]. Moreover, the plasma transaminase concentration of ALT and AST (Fig 4B) as well as hepatic free cholesterol content (Fig 1B) followed the same regulation pattern. Therefore, the steatosis we observed in wild-type mice fed the OLIV diet is not associated with hepatic damage and, strikingly, the deficiency of LXR revealed liver injury induced by the OLIV diet. These results are in accordance with two studies in which the loss of function of key lipogenic genes, *Scd1* [49] and *Dgat2* [50], in a methionine and choline deficient diet-induced NASH model, exacerbates hepatic damages despite reduced steatosis. LXR also appears to display putative protective role in NASH (reviewed in [51]). It has been also reported that lipogenesis induced by ChREBP overexpression dissociates hepatic steatosis from insulin resistance a feature linked to NAFLD [13]. More generally, it appears that lipogenesis may lead to the production of signaling molecules decreasing disease risks, a concept called “lipoexpediency” [52].

Finally, we also observed that the oleate rich diet drives beneficial effects on cholesterol parameters. Indeed, OLIV diet decreased the cholesterolemia in wild-type mice although no changes occurred in LXR knockout mice (Fig 4C). It is interesting to note the antinomy existing between cholesterol and oleic acid. Indeed, we showed that oleate rich diet decreased cholesterolemia and it has also been reported that oleate increases the expression of *ABCA1*, a key player involved in reverse cholesterol transport, in human neutrophils [14]. Conversely, *Scd1* whole body knockout mice fed a low fat high carbohydrate diet (a model of complete oleic acid depletion) displays hypercholesterolemia and cholestasis [53]. Therefore, it seems that oleic acid regulates cholesterol content in the organism. Cholesterol is an important factor in the progression of several diseases such as cardiovascular diseases [54,55], cancers [56–59], Alzheimer’s disease [60] and NASH [28,61], thus, its control by oleate appears to be of importance. Here, we showed that LXR mediates the decrease of cholesterolemia in response to an oleate-rich diet. This role of LXR on cholesterolemia could explain the beneficial effects of olive oil in epidemiological studies (reviewed in [1]). However, this potent link needs further examination as well as the mechanisms by which LXR activity is triggered in response to dietary oleic acid. Indeed, unlike essential fatty acids of the n-6 and n-3 series, oleic acid does not act as an inhibitor of lipogenic enzymes. Therefore, it is possible that increasing the relative abundance of dietary oleate reduces the effects of dietary polyunsaturated fatty acids on gene expression [15;16;62]. In addition, the hypothesis that oleate directly binds and activates LXR is unlikely. Indeed, the work of Ou *et al*. showed that, in cell culture oleic acid represses LXR activity [15]. Moreover, it has recently been demonstrated that unlike short chain fatty acids, oleic acid is unable to bind and activate LXRα [63]. In addition, growing evidences highlight the modulation of co-activators [64] and regulators of nuclear receptors activity such as SIRT1 [45] by oleic acid. Such indirect mechanisms can be considered in the regulation of LXR activity by oleic acid. It is also possible that LXR may regulate key factors in the well-described response to exogenous oleic acid on hepatic lipogenesis and inflammation that has been highlighted in vivo [9;64].

Altogether, our work further suggests that oleic acid may play some important function in the regulation of gene expression. We also present evidence that LXR is involved in this process. The elucidation of underlying mechanisms requires further investigation.

## Supporting information

S1 Table Diets composition. (DOCX)

S2 Table Relative fatty acid composition of dietary oils (%). (DOCX)

S3 Table Oligonucleotide sequences for real time PCR. (DOCX)

S4 Table Fatty acids were analyzed by gas chromatography. Data are the mean of the masse percentage measured in liver of LXR+/+ and LXR-/- mice fed the REF or the OLIV diet. ^a^ Significant genotype effect. ^b^ Significant difference versus REF diet. (n = 6 animals per group).
(DOCX)

S1 Fig Quantification of cytoplasmic and nuclear proteins presented in Fig 3. **(A)** Cytoplasmic ACLY, P-ACLY, ACC, FAS, ELOVL6, SCD1, SREBP-1 and ChREBP protein quantification. (B) Nuclear SREBP-1, ChREBP and LXR protein quantification. Values are mean ± SEM (n = 6); a Significant gentotype effect. b Significant difference versus REF diet.
(TIF)

S2 Fig Oleate does not induce LXR target genes *in vitro*. mRNA expression of Fatty acid synthase (Fasn) and Stearoyl-CoA Desaturase 1 (Scd1) measured by qPCR in Hepa1 cells (A) and in Mouse embryonnic fibroblast (B) treated with TO901317 or with Oleic Acid (OA) provided as an albuminic complex. Values are means ± SEM (n = 3), *: Significant (p<0,05) effect compared with DMSO (for TO901317 treated conditions) or with BSA (for OA treated conditions).
(TIF)

S3 Fig Triolein promotes lipogenic gene expression in vivo. Hepatic *Acaca*, *Fasn*, *Elovl6*, *Scd1* and *Cyp4a14* mRNA levels quantified by qPCR. Data are the mean ± SEM of values measured in C57Bl/6J fed fat free diet and daily gavaged with triolein (TRIOL), the same olive oil we used in the OLIV diet (OLIV), a mix of theses two oils (OLIV/TRIOL) and a mix of OLIV and fish oil (OLIV/FISH) as a negative control for lipogenic genes. a Significant difference versus OLIV/FISH, b Significant difference versus OLIV, c Significant difference versus OLIV/TRIOL (n = 6 per group except OLIV/FISH group (n = 4)).
(TIF)

## Abbreviations

ChREBP: Carbohydrate Response Element Binding Protein
FASN: Fatty acid synthase
LXR: Liver X Receptor
NAFLD: Non Alcoholic Fatty Liver Disease
NASH: Non Alcoholic Steatohepatitis
SCD1: Stearoyl-CoA desaturase 1
SREBP-1c: Sterol Responsive Element Binding Protein-1c
