# Supplementary material for: Dietary oleic acid regulates hepatic lipogenesis through a liver X receptor-dependent signaling
Source: PLoS One. 2017 Jul 21;12(7):e0181393. doi: 10.1371/journal.pone.0181393 (PMC5521785; doi:10.1371/journal.pone.0181393)
Supplement: S1 Table — (DOCX) [file pone.0181393.s001.docx]

**S1 Table**: Diets composition

| Components | % (w/w) |
| --- | --- |
| Cellulose | 2 |
| Casein | 22 |
| Starch | 43.6 |
| Methionin | 0.2 |
| Sucrose | 21.8 |
| Minerals | 4.5 |
| Vitamins | 1 |
| Oil | 5 |
