# Supplementary material for: Dietary oleic acid regulates hepatic lipogenesis through a liver X receptor-dependent signaling
Source: PLoS One. 2017 Jul 21;12(7):e0181393. doi: 10.1371/journal.pone.0181393 (PMC5521785; doi:10.1371/journal.pone.0181393)
Supplement: S2 Table — (DOCX) [file pone.0181393.s002.docx]

**S2** **Table**: Relative fatty acid composition of dietary oils (%)

| Diet | REF | OLIV |
| --- | --- | --- |
| C12:0 | 0 | 0 |
| C14:0 | 0 | 0 |
| C16:0 | 5.9 | 9.6 |
| C18:0 | 2.8 | 3.3 |
| C18:1 n-9 | 39.7 | 78.9 |
| C18:2 n-6 | 47 | 7.6 |
| C18:3 n-3 | 4.6 | 0.6 |
| C20:5 n-3 | 0 | 0 |
| C22:6 n-3 | 0 | 0 |
