# Supplementary material for: Dietary oleic acid regulates hepatic lipogenesis through a liver X receptor-dependent signaling
Source: PLoS One. 2017 Jul 21;12(7):e0181393. doi: 10.1371/journal.pone.0181393 (PMC5521785; doi:10.1371/journal.pone.0181393)
Supplement: S3 Table — (DOCX) [file pone.0181393.s003.docx]

| **S3 Table**: Oligonucleotide sequences for real time PCR | | |  |
| --- | --- | --- | --- |
|  |  |  |  |
| **Gene** | **NCBI Refseq** | **Forward primer (5'-3')** | **Reverse primer (5'-3')** |
| *Abca1* | NM_013454 | GCGCTACAACATGGACATCCT | GCTGGGTCGGGAGATGAGA |
| *Abcb1a* | NM_011076 | CATGACAGATAGCTTTGCAAGTGTAG | GGCAAACATGGCTCTTTTATCG |
| *Abcc2* | NM_013806 | AAGCAGATTGACACCAACCAGAA | GGCCGAGCAGAAGACAATCA |
| *Abcg5* | NM_031884 | TCGCCACGGTCATTTTCA | GCCAAAAGAGCAGCAGAGAAATA |
| *Abcg8* | NM_026180 | ATCCATTGGCCACCCTTGT | GCGTCTGTCGATGCTGGTC |
| *Acaca* | NM_133360 | TTACAGGATGGTTTGGCCTTTC | CAAATTCTGCTGGAGAAGCCAC |
| *Acacb* | NM_133904 | CCTGAATCTCACGCGCCTA | CAGATGGAGTCCAGACATGCTG |
| *Acat1* | NM_144784 | ATTACTCCCATCACCATCTCAGTG | CACGGTCTTGAGCTTTGGC |
| *Acat2* | NM_009338 | GTTATGGGAGTAGGACCAATTCCAG | TCCAAGTTCTTTAGCTATTGCCG |
| *Acly* | NM_134037 | AAAGCTTGGCCTCGTCGG | GGGACGAAGGGTTCAATGAGA |
| *Acox1* | NM_015729 | CAGACCCTGAAGAAATCATGTGG | CAGGAACATGCCCAAGTGAAG |
| *Agpat1* | NM_018862 | CATGAGGCTAGAAGGGAAGACG | CCGGGCCACAGCTCC |
| *Agpat2* | NM_026212 | CGCACCGTGGATAACATGAG | CTCTGGTGATTAGAGATGATGACACA |
| *Agpat6* | NM_018743 | AGCTTTGAAATTGGAGCCACTG | CAATGGCCCAACTGGTCATC |
| *ApoA1* | NM_009692 | TGGGCCAACAGCTGAACC | TCCCAGAAGTCCCGAGTCAA |
| *ApoB* | NM_009693 | AATCTGTGGTTTCATCATGAGGAC | GGCCAGCTTGAGTTCGTACCT |
| *ApoE* | NM_009696 | TTGGTCACATTGCTGACAGGAT | GAGTGGCAAAGCAACCAACC |
| *Ascl3* | NM_001033606 | TGGACTGAATGAGACAGAGGTGA | CAGACGTGGGACCAAAGAGACTA |
| *Atf4* | NM_009716 | ATGGGTTCTCCAGCGACAAG | CCGGAAAAGGCATCCTCC |
| *Atp5b* | NM_016774 | AAGGATCACCACCACCAAGAAG | CAAATGGGCAAAGGTGGTTG |
| *Bien* | NM_023737 | CGTCTCCTCGGTTGGTGTTC | ATTATCTTCTTTGCAGTATCTAGCTGCTT |
| *Car* | NM_009803 | GCTGCAAGGGCTTCTTCAGA | CCTTCCAGCAAACGGACAGA |
| *Ccl2* | NM_011333 | GGTGTCCCAAAGAAGCTGTAGTTTT | AGTTGTAGGTTCTGATCTCATTTGGTT |
| *Cd36* | NM_007643 | GTTAAACAAAGAGGTCCTTACACATACAG | CAGTGAAGGCTCAAAGATGGC |
| *Cept1* | NM_133869 | GAAACAGGCCAGAAGAACTAACAGTA | GCAATACAAGTTCCAAGAACCACA |
| *Chka* | NM_013490 | AGGCCTTCGCGAGGACC | CAACGCTGGCTATGGAGTCTG |
| *Chrebp* | NM_021455 | ACTCAGGGAATACACGCCTACAG | GAAGAAGGAATTCAGAGCTCAGAAA |
| *Cnr2* | NM_009924 | CAAGGCTCCACAAGACCCTG | GTTGGTCACTTCTGTCTCCCG |
| *Cpt1a* | NM_013495 | GAAGAAGAAGTTCATCCGATTCAAG | GATATCACACCCACCACCACG |
| *CrebH* | NM_145365 | CATTGATGGTCTGGAGAACCG | TTGCTGGTTGACTGGACCAC |
| *Cyb5r3* | NM_029787 | GAGTAGCGGGTGTAGAACCGTG | TCATGAAGAGACTGTAGATGAACCAGA |
| *Cyp27a1* | NM_024264 | CTGCACTTGCCCGACCTC | CACTAGCCAGATTCACATTGGTGT |
| *Cyp2b10* | NM_009999 | TTTCTGCCCTTCTCAACAGGAA | ATGGACGTGAAGAAAAGGAACAAC |
| *Cyp2c29* | NM_007815 | GCTCAAAGCCTACTGTCA | CATGAGTGTAAATCGTCTCA |
| *Cyp39a1* | NM_018887 | ATGCAGTCTTCTGGACCCATTAC | CAAGCTCTTTGTTTATATTCAATCCG |
| *Cyp3a11* | NM_007818 | TCACACACACAGTTGTAGGCAGAA | GTTTACGAGTCCCATATCGGTAGAG |
| *Cyp46a1* | NM_010010 | AGTATGGTCCTGTTGTAAGAGTCAATGT | ACATCTTGGAGTCCTTGTTGTACTTG |
| *Cyp4a14* | NM_007822 | TCAGTCTATTTCTGGTGCTGTTC | GAGCTCCTTGTCCTTCAGATGGT |
| *Cyp51a* | NM_020010 | TGGCTGCCTCTGCCAAGT | GGTTCTTTTGACAGCCTGCG |
| *Cyp7a1* | NM_007824 | AGAGCAACTAAACAACCTGCCAGTA | GCACTGGAGAGCCGCAGA |
| *Cyp7b1* | NM_007825 | ACATGGTGACACTTTCACTGTCTTC | GAACTTCTGAAAGCTTAATTGTTTTGG |
| *Dgat1* | NM_010046 | GGACCAGCGTGGGCG | AAGAATCTTGCAGACGATGGC |
| *Dgat2* | NM_026384 | ACAGCTGCAGGTCATCTCAGTACTA | AGCACAGCTATCAGCCAGCA |
| *Dhcr24* | NM_053272 | ATATCTACTACTACGTGCGCGCC | CTGCCCTGTTCCTTCCATTC |
| *Dhcr7* | NM_007856 | GACCCTCATTAACCTGTCCTTCG | CCAGGTTTCATTCCAGAAGAAGTC |
| *Ebp* | NM_007898 | TGATCGAGGGCTGGTTCTCT | ATATCGGCTATCTCCCTTGGAATA |
| *Eci* | NM_010023 | GTTCACCATCAGCCTGGAGAAG | AGAAGATACCCGGGCATTCC |
| *Edem1* | NM_138677 | AAGCCCTCTGGAACTTGCG | ATGGCCTGTCTGGATGTTCAC |
| *Elovl1* | NM_019422 | GTACCTACACCTGGCGCTGTG | CAGGCCACTCGAACCATCC |
| *Elovl2* | NM_019423 | CAGCTGGGAAGGAGGTTACAACT | AATCGTGTCCAGGAACTCCACTA |
| *Elovl3* | NM_007703 | CGTAGTCAGATTCTGGTCCT | CCAGAAGAAGTGTTCCGTTG |
| *Elovl5* | NM_134255 | TCGATGCGTCACTCGTACCTATT | ATTTTGGTCCCAGCCATACAAT |
| *Elovl6* | NM_130450 | TCTGATGAACAAGCGAGCCA | TGGTCATCAGAATGTACAGCATGT |
| *Fabp* | NM_017399 | GGCAAGTACCAATTGCAGAGC | AGGTCCTCGGGCAGACCTA |
| *Fads1* | NM_146094 | TCAACATGCACCCCCTCTTC | GATGGTTGTATGGCATGTGCTT |
| *Fads2* | NM_019699 | TCCAGTACCAGATCATCATGACAA | GGTGTAGAAGAAACGCATATAGTAGCTG |
| *Fasn* | NM_007988 | AGTCAGCTATGAAGCAATTGTGGA | CACCCAGACGCCAGTGTTC |
| *Fdft1* | NM_010191 | AGGAGTTCTATAACCTGCTGCGAT | GGTCTTCAAGCTGCTGCTGAGT |
| *Fdps* | NM_134469 | TGCTATTGCCCGGCTCA | ATCCTGTTTCTTCGGCTCCA |
| *Fgf21* | NM_020013 | AAAGCCTCTAGGTTTCTTTGCCA | CCTCAGGATCAAAGTGAGGCG |
| *Fgfr4* | NM_008011 | GTATGGATCCCTCCCGGC | GGTCTGCCAAATCCTTGTCG |
| *Fsp27* | NM_178373 | CATGAAGTCTCTCAGCCTCCTGTA | CAGCTGTTGGGTCACCACTG |
| *Fxra* | NM_009108 | CCACCGGCTGTCAGGATT | CGCGTGTTCTGTTAGCATACCTT |
| *G6pc* | NM_008061 | CTCACTTTCCCCACCAGGTC | GCTGAAAGTTTCAGCCACAGC |
| *Ggps* | NM_010282 | AAGCTGAGAGGATTCTTCTAGAGCC | TGAAAGTTTGCTTCTCACCTGTTTA |
| *Glut2* | NM_031197 | TTTGCAGTGGGCGGAATGG | GCCAACATTGCTTTGATCCTT |
| *Gpat* | NM_0081449 | AGACGAAGCCTTCCGACGA | TGGACATGATAGCGCAGGACT |
| *Grp78* | NM_022310 | GCCGAGGAGGAGGACAAGA | TCTTGAACACACCGACGCA |
| *Hmgcr* | NM_008255 | CTTGTGGAATGCCTTGTGATTG | GAAGAATGTCATGAACACAAAGTAGTTG |
| *Hmgcs1* | NM_145942 | CCTGGACCGCTGCTATTCTG | TGAAAGATCATGAAGCCAAAATCA |
| *Hmgcs2* | NM_008256 | TGCAGGAAACTTCGCTCACA | AAATAGACCTCCAGGGCAAGGA |
| *Hnf4a* | NM_008261 | CTTGGAGCCACCAAGAGGTC | CTAGCTCTGGACAGTGCCGAG |
| *Hsd17b7* | NM_010476 | ACTTCGGTGCAGGGCGT | CACAGGTGGAGGTCATCGTCT |
| *Idol* | NM_153789 | CCGAGCCATCACCGAAAC | TCATGACGGCACTGGTGACT |
| *Insig1* | NM_153526 | GAGGTGTCACAGTGGGAAACATAG | TCTTCATCACACCCAGGACCA |
| *Insig2* | NM_133748 | TGTATATTTTTTGCTGGAGGCATAAC | TTCAGCAATAACTTTGCATTCATACAT |
| *Lpk* | NM_013631 | TCGACTCAGAGCCTGTGGC | AGTCGTGCAATGTTCATCCCT |
| *Ldlr* | NM_010700 | GCAAGGACATGAGCGACGA | CTCCCCACTGTGACACTTGAAC |
| *Lipin1* | NM_172950 | ATGTTTCCCATAGAGATGAGCTCG | GAATGGTGGTACATCATTAGGAAGAG |
| *Lipin2* | NM_001164885 | AGGACAATAGGAAGGAGGAGCAG | TTGTAGTCCTCTTCCTTTAAGGAAGC |
| *Lipin3* | NM_022883 | CCCTCTGGGCATCCACAA | TGGCCCCCATCCCATACT |
| *Lpcat3* | NM_145130 | TACAAGGACAGCTACCTCATCCATC | GAAGCACGACACATAGCAAGGA |
| *Lpl* | NM_008509 | ATGGCAAGCAACACAACCAG | TGTGGAAACCTCGGGCAG |
| *Lss* | NM_146006 | ATGAGTTGGGTCGGCAGAGAT | GCGCTTTTGGTAAGTCCGTG |
| *Me1* | NM_008615 | CATTCGAGGCGTTTCGTTG | CAGGTAGGATCTGGTCATAATTAGTGC |
| *Mmp9* | NM_013599 | CTCGAGGGCTTCCCTCTGA | GGCTGGAGGCCTTGGGT |
| *Mttp* | NM_008642 | TCAGGAAGCTGTGTCAGAATGAAG | TTTCAAGTCCTCCCAGGATCA |
| *Mvd* | NM_138656 | CGGTCAACATCGCAGTTATCAA | GTGCAGCGTGACGCTCAG |
| *Mvk* | NM_023556 | GCTTCAGCGACTGGACACG | ACAGGTAGAGAAAGGCAAGCAGA |
| *Npc1l1* | NM_207242 | ATCACCTTGCTGGGTCTGCTAC | GTACTGTGGGCAAGAAGGCTCT |
| *Nsdhl* | NM_010941 | TGCTGGAGCGAGGCTATACTG | CAGTGGAAAACTGTGCTTACACCTT |
| *P2ry13* | NM_028808 | CTGAGTCTCTTCCAAAACAAAGCTG | ACCGCTCAGACTTGTTGAAGC |
| *P2yr1* | NM_008772 | GCACGAGATCCTAGCTCCTGA | GCACACACTGGTCTTTTGGTCA |
| *Pcsk9* | NM_153565 | AGGAAGACCGCTCCCCTG | TGGTATCTAAGAGATACACCTCCACCT |
| *Pdk4* | NM_013743 | ATCGCCAGAATTAAACCTCACAC | TGGATTGGTTGGCCTGGA |
| *Pepck* | NM_011044 | GAACCCCAGCCTGCCC | GAGCAACTCCAAAAAACCCG |
| *Pgc1b* | NM_133249 | CTTTGCGGCACGGCAG | CTGGGCTGAGCTTGGTGTCT |
| *Plin3* | NM_025836 | GGCTGGACAGACTGCAGGA | TCTTGAGCCCCAGACACTGTAG |
| *Plin5* | NM_025874 | CGCTCCATGAGTCAAGCCA | CTCAGCTGCCAGGACTGCTA |
| *Pltp* | NM_011125 | GGATTAAAGTGTCCAATGTCTCCTG | GTGGAGAAAAAGTTATACATCCTCCTG |
| *Pmdci* | NM_016772 | GGAAAGATGTTCACTTCAGGTATTGAC | CGGGCCGCATCATCTC |
| *Pmvk* | NM_026784 | GGAAGGCGTGTCCCAGC | GCCCCATAGGCCTCCTGA |
| *Pnpla3* | NM_054088 | ACGCGGTCACCTTCGTGT | AGCCCGTCTCTGATGCACTT |
| *Ppara* | NM_011144 | CCCTGTTTGTGGCTGCTATAATTT | GGGAAGAGGAAGGTGTCATCTG |
| *Pparg1* | NM_011146 | CCACCAACTTCGGAATCAGCT | TTTGTGGATCCGGCAGTTAAGA |
| *Pparg2* | NM_011146 | ATGGGTGAAACTCTGGGAGATTCT | CTTGGAGCTTCAGGTCATATTTGTA |
| *Pxr* | NM_010936 | AGAGATCATCCCTCTTCTGCCAC | GATCTGGTCCTCAATAGGCAGGT |
| *Reverba* | NM_145434 | CAGCTGGTGAAGACATGACGAC | GGAGGAGCCACTAGAGCCAA |
| *Sc4mol* | NM_025436 | AAGCCATCTATTTCTTGTTCTCTTTACCT | CAAACACTTCCACTGGCCTTC |
| *Sc5d* | NM_172769 | CAGCATCCCCACCGTCTC | AAAAGAGGAAGGATACGACGCTAA |
| *Scarb* | NM_016741 | TCCCTCATCAAGCAGCAGGT | ACCTCGTTTGGGTTGACCAC |
| *Scd1* | NM_009127 | CAGTGCCGCGCATCTCTAT | CTGACTGGCAAATATAGCTGTATCCT |
| *Scd2* | NM_009128 | CCCCTACGACAAGAACATTAGC | GGTAGTTGTGGAAGCCCTCG |
| *Sec14l1* | NM_028777 | TCCTTGTCCCAGATGCTGCT | GATGTAGTCGGCATCTAATTTATCGT |
| *Shp* | NM_011850 | CCCAAGGAGTATGCGTACCTGA | TGTGCGATGTGGCAGGAG |
| *Sirt1* | NM_019812 | GCTGTGAAGTTACTGCAGGAGTGT | CCGCAAGGCGAGCATAGATA |
| *Soat1* | NM_009230 | TGTTGGCAGCAGAGGCG | GGTCACAAAGTCATCGAAGTGG |
| *Spot14* | NM_009381 | AACGGAGGAGGCCGAAGAAG | GTTGATGCACCTCGGGGTCT |
| *Sqle* | NM_009270 | GGAGGCTACCGTGTTCTCCA | CTGCACTTGGTTGGTTTCTGAC |
| *Srebp1a* | NM_011480 | CAGACACTGGCCGAGATGTG | CTTGGTTGTTGATGAGCTGGAG |
| *Srebp1c* | NM_011480 | CAGACACTGGCCGAGATGTG | CTTGGTTGTTGATGAGCTGGAG |
| *Srebp2* | NM_033218 | GTACTGCGCCCAGAGGAGC | GCCTGAGGTTTCACCAAGGAC |
| *Star1* | NM_011485 | AAGGCCTTGGGCATACTCAAC | TGGCACCATCTTACTTAGCACTTC |
| *Sult1e1* | NM_023135 | ATTTCACTTCTTCCACGGGAAC | CCAAAAACTTCATAATACTCAGGCATAG |
| *Tbp* | NM_013684 | ACTTCGTGCAAGAAATGCTGAA | GCAGTTGTCCGTGGCTCTCT |
| *Tm7sf2* | NM_028454 | AAGGCCTGGAACTGAAGGACA | ACCAGAGCCTGGAAGCCAT |
| *Trb3* | NM_175093 | GGCCTTATATCCTTTTGGAACGA | CACCGCCTGGGCCTC |
| *Ubxd8* | NM_178397 | CCAGCTCGGCCCCTACA | GTAATAACCCCATCCAAGCAGG |
| *Ucp2* | NM_011671 | CATGGTAGCCACCGGCA | CTTCAATCGGCAAGACGAGAC |
| *Ugt1a2* | NM_201645 | TCCTTCCTCTTATATTCCGAACCTAC | GAAAACAACGATGCCATGCTC |
