# Supplementary material for: Dietary oleic acid regulates hepatic lipogenesis through a liver X receptor-dependent signaling
Source: PLoS One. 2017 Jul 21;12(7):e0181393. doi: 10.1371/journal.pone.0181393 (PMC5521785; doi:10.1371/journal.pone.0181393)
Supplement: S4 Table — Data are the mean of the masse percentage measured in liver of LXR+/+ and LXR-/- mice fed the REF or the OLIV diet. a Significant genotype effect. b Significant difference versus REF diet. (n = 6 animals per group). (DOCX) [file pone.0181393.s004.docx]

**S4 Table** : Fatty acids were analyzed by gas chromatography (n=6 animals per group). Data are the mean of the masse percentage measured in liver of LXR+/+ and LXR-/- mice fed the REF or the OLIV diet. ^a^ Significant genotype effect. ^b^ Significant difference versus REF diet.

|  | LXR+/+ | | LXR-/- | |
| --- | --- | --- | --- | --- |
|  | REF | OLIV | REF | OLIV |
| C14:0 | 0.922 | 0.931 | 1.209 | 1.195 |
| C14:1 n-7 | 0.027 | 0.029 | 0.373^a^ | 0.314^a^ |
| C16:0 | 15.678 | 14.246 | 12.629^a^ | 12.349 |
| C16:1 n-9 | 0.696 | 0.871^b^ | 0.463^a^ | 0.581a |
| C16:1 n-7 | 9.998 | 10.492 | 18.642^a^ | 19.200^a^ |
| C18:0 | 1.146 | 1.023 | 0.671^a^ | 0.725^a^ |
| C18:1 n-9 | 41.361 | 58.519^b^ | 32.025^a^ | 45.202^a,b^ |
| C18:1 n-7 | 3.287 | 4.173^b^ | 2.601^a^ | 2.697^a^ |
| C18:2 n-6 | 24.606 | 8.193^b^ | 28.779 | 16.121^a,b^ |
| C18:3 n-3 | 1.271 | 0.567^b^ | 1.989^a^ | 1.038^b^ |
| C20:1 n-9 | 0.957 | 0.963 | 0.620 | 0.576 |
| C20:4 n-6 | 0.052 | 0 | 0 | 0 |
| n-6/n-3 | 19.890 | 13.004 | 14.731 | 15.652^a^ |
