# Supplementary figures and images for: Dietary oleic acid regulates hepatic lipogenesis through a liver X receptor-dependent signaling
Source: PLoS One. 2017 Jul 21;12(7):e0181393. doi: 10.1371/journal.pone.0181393 (PMC5521785; doi:10.1371/journal.pone.0181393)

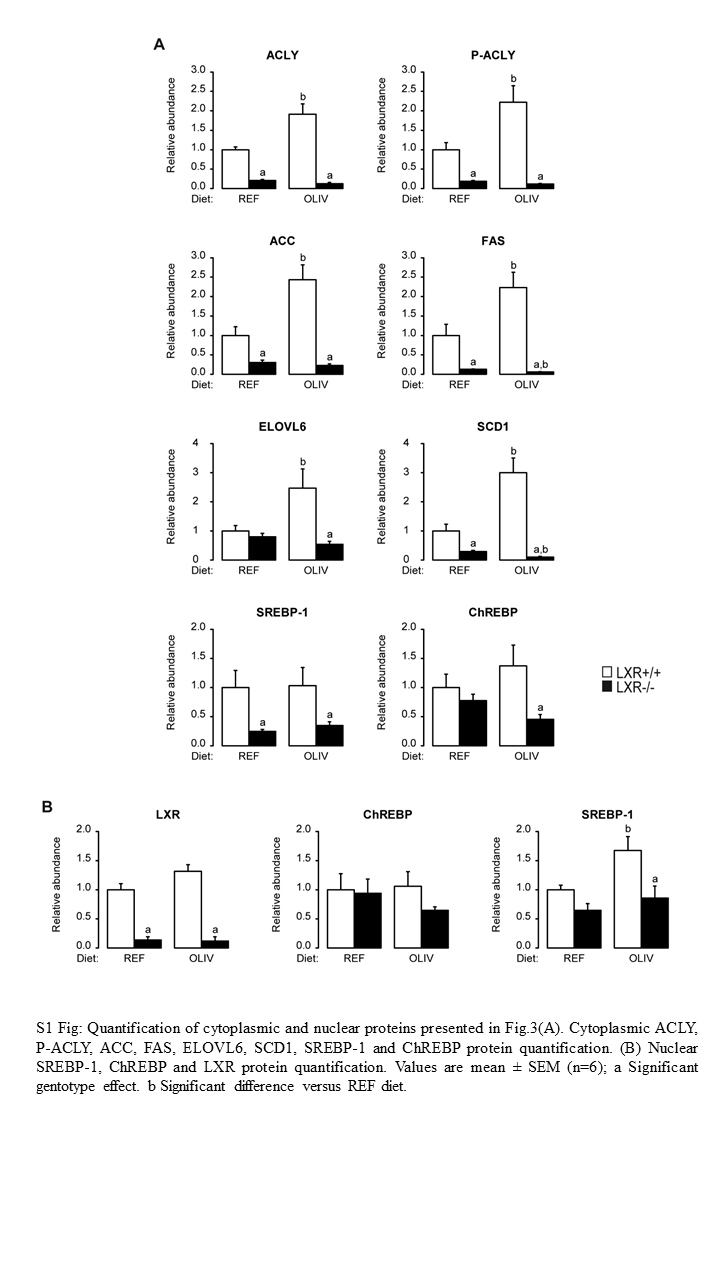

Supplement: S1 Fig — (A) Cytoplasmic ACLY, P-ACLY, ACC, FAS, ELOVL6, SCD1, SREBP-1 and ChREBP protein quantification. (B) Nuclear SREBP-1, ChREBP and LXR protein quantification. Values are mean ± SEM (n = 6); a Significant gentotype effect. b Significant difference versus REF diet. (TIF) [file pone.0181393.s005.tif]

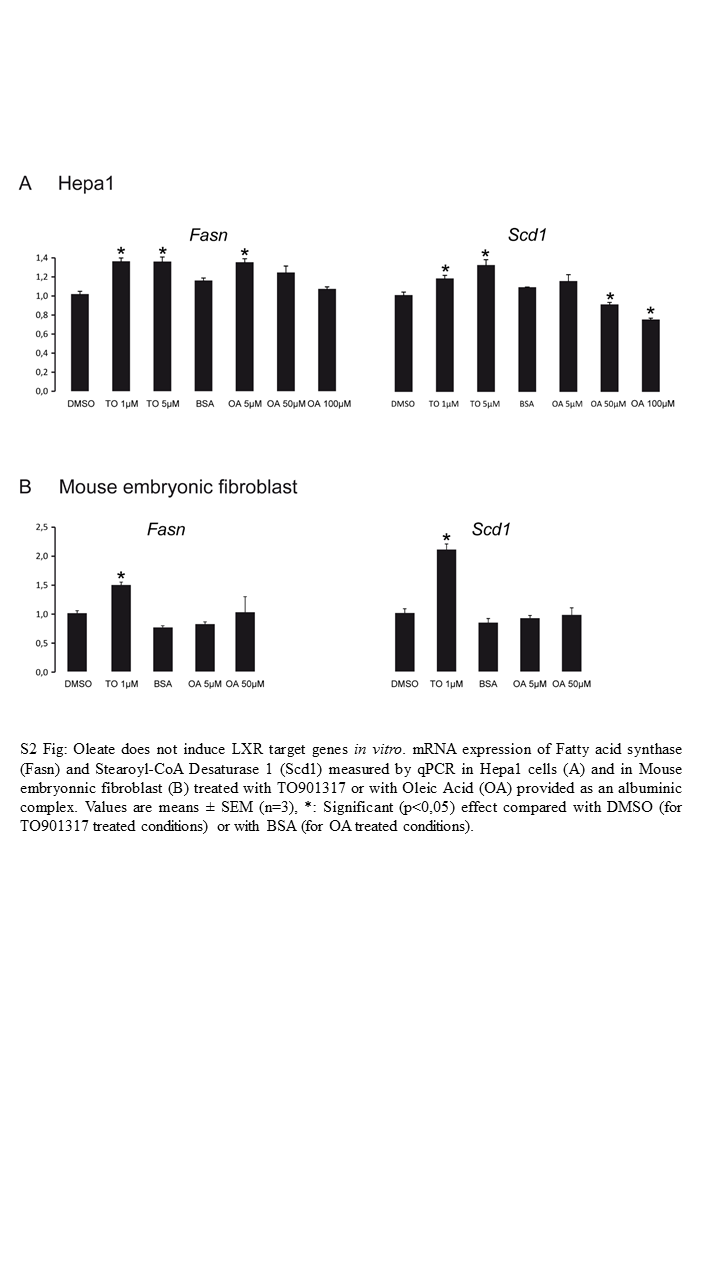

Supplement: S2 Fig — mRNA expression of Fatty acid synthase (Fasn) and Stearoyl-CoA Desaturase 1 (Scd1) measured by qPCR in Hepa1 cells (A) and in Mouse embryonnic fibroblast (B) treated with TO901317 or with Oleic Acid (OA) provided as an albuminic complex. Values are means ± SEM (n = 3), *: Significant (p<0,05) effect compared with DMSO (for TO901317 treated conditions) or with BSA (for OA treated conditions). (TIF) [file pone.0181393.s006.tif]

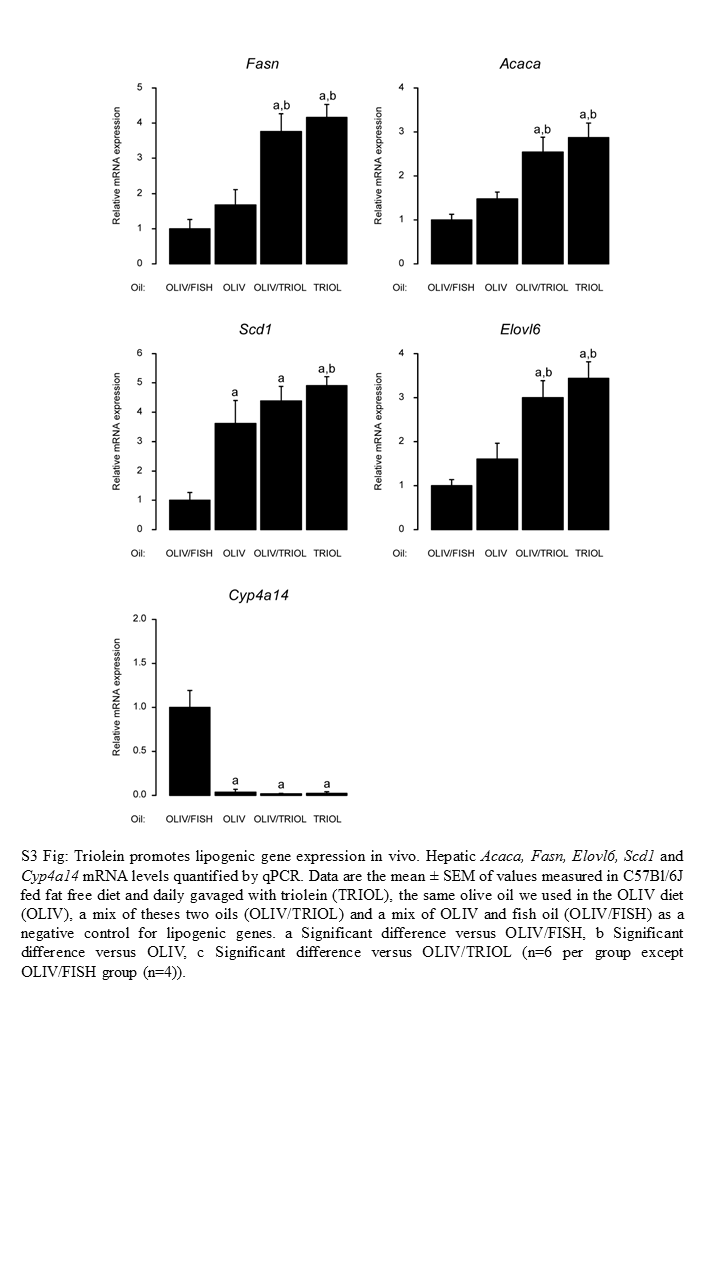

Supplement: S3 Fig — Hepatic Acaca, Fasn, Elovl6, Scd1 and Cyp4a14 mRNA levels quantified by qPCR. Data are the mean ± SEM of values measured in C57Bl/6J fed fat free diet and daily gavaged with triolein (TRIOL), the same olive oil we used in the OLIV diet (OLIV), a mix of theses two oils (OLIV/TRIOL) and a mix of OLIV and fish oil (OLIV/FISH) as a negative control for lipogenic genes. a Significant difference versus OLIV/FISH, b Significant difference versus OLIV, c Significant difference versus OLIV/TRIOL (n = 6 per group except OLIV/FISH group (n = 4)). (TIF) [file pone.0181393.s007.tif]
